# Nesfatin-1 ameliorates blood-brain barrier dysfunction in Alzheimer’s disease by targeting VEGF-R1 and reducing cellular senescence in brain vascular endothelial cells

**DOI:** 10.1038/s41398-025-03528-8

**Published:** 2025-09-03

**Authors:** Biyue Zhang, Shumei Zhang, Zeming Guo, Chunzhan Hong, Futian Zhang, Huasong Lin

**Affiliations:** 1 Department of Neurology, the Second Affiliated Hospital of Fujian Medical University, Quanzhou, Fujian 362000 China; 2 Department of Implant Dentistry III, Stomatological Hospital of Xiamen Medical College, Xiamen, Fujian 361006 China; 3 Department of Neurology, Nan’an Hospital, Quanzhou, Fujian 362300 China; 4 Department of Geriatric Medicine, the Seventh Affiliated Hospital of Sun Yat-sen University, Shenzhen, Guangdong 518107 China

**Keywords:** Physiology, Molecular neuroscience

## Abstract

Cellular senescence and associated endothelial permeability are crucial factors in the dysfunction of the blood-brain barrier (BBB) in neurodegenerative diseases, including Alzheimer’s disease (AD). Nesfatin-1 (NF-1), a neuropeptide involved in regulating appetite and energy homeostasis, has not been extensively studied for its pathophysiological role in AD. In this study, we found that NF-1 treatment improved cellular senescence in brain vascular endothelial bEnd.3 cells by restoring the expression of hTERT and TERF2 against oligomerized Aβ_1-42_. Additionally, NF-1 reduced p53 and p21 protein levels in bEnd.3 cells exposed to oligomerized Aβ_1-42_. Notably, NF-1 reduced oligomerized Aβ_1-42_-induced endothelial monolayer permeability by maintaining transendothelial electric resistance (TEER) and the levels of tight junction proteins claudin 5 and ZO-1. Furthermore, NF-1 suppressed the expression of VEGF-R1 but not VEGF-R2 in bEnd.3 cells exposed to oligomerized Aβ_1-42_. Overexpression of VEGF-R1 negated the protective effects of NF-1 against oligomerized Aβ_1-42_-induced cellular senescence and increased endothelial monolayer permeability, indicating the involvement of VEGF-R1 in this process. Using a transgenic (Tg APPswe/PSEN1dE9) AD mouse model, we demonstrated that NF-1 administration lowered VEGF-R1 expression in the brain cortex of AD mice. Moreover, NF-1 mitigated BBB dysfunction and enhanced the expression of claudin 5 and ZO-1 in the brains of AD mice. Our results suggest that NF-1 may be a potential therapeutic strategy for treating AD.

## Introduction

Alzheimer’s disease (AD) is characterized by a decline in cognitive abilities and is a common neurodegenerative condition. Its clinical manifestations include decreased cognitive function, memory impairment, emotional control deficits, and motor disabilities [1]. AD affects 33 million people worldwide, with the number of patients expected to reach 150 million by 2050, making it the fourth leading cause of death after cardiovascular diseases, stroke, and cancer. Risk factors for AD include advanced age, and over 60% of patients require care, resulting in a significant socioeconomic burden [2].

The blood-brain barrier (BBB) is a protective mechanism of the brain, and its integrity is crucial for maintaining brain health by restricting the entry of harmful substances and regulating the exchange of essential nutrients [3]. Brain endothelial cells (BECs) are key components in the formation of the BBB, maintaining the integrity and selective permeability of the brain’s microvasculature through tight cellular connections [4]. As age progresses, BECs may undergo senescence, altering their function and structure, which leads to BBB damage. Senescent BECs exhibit reduced proliferative capacity and increased inflammatory responses, and their cellular connections may become loose, allowing substances from the bloodstream to penetrate brain tissue more easily. Xu et al. (2023) have shown that cellular senescence is associated with significant changes in telomere maintenance and telomerase activity, which can impact the integrity of cellular structures and contribute to age-related diseases [5]. A damaged BBB fails to effectively prevent harmful substances and pathogens from entering the brain [6, 7].

Amyloid-β _1-42_ (Aβ_1-42_) oligomers are toxic β-amyloid proteins closely associated with AD [8]. The accumulation of Aβ_1-42_ in the brain not only directly damages neurons but may also lead to the destruction of the BBB by affecting the function of BECs [9]. Specifically, Aβ_1-42_ oligomers interact with vascular endothelial growth factor receptor 1 (VEGF-R1), activating a series of signal transduction pathways that induce senescence in BECs [10], which affects the proliferative and reparative abilities of the endothelial cells and may lead to an increase in inflammatory factors they secrete, further damaging the health of the cerebral vasculature [11]. Furthermore, Aβ_1-42_ oligomers, through the action of VEGF-R1, reduce the expression of tight junction (TJ) proteins ZO-1 and claudin-5. These proteins are crucial for maintaining the integrity of the BBB. The reduction of these proteins results in loosened cellular connections, weakening the barrier function of the BBB. As a consequence, substances from the bloodstream can penetrate the brain more easily, exacerbating neuroinflammation and neurodegenerative changes [12]. Therefore, repairing the damage to the BBB induced by Aβ_1-42_ may become an important strategy in the treatment of AD.

Nesfatin-1 (NF-1) is derived from its precursor protein, nucleobindin 2, through the specific cleavage by prohormone convertases at certain sites [13], which is a peptide with multiple roles, including regulating fat metabolism, food intake, sleep, mental activity, gastrointestinal motility, and glucose metabolism [14–17]. In neurological disorders, NF-1 can counteract the damage and apoptosis of dopaminergic neurons induced by MPP(+)/MPTP through the activation of the C-Raf-ERK1/2 dependent anti-apoptotic signaling pathway. This offers a new potential target for treating neurodegenerative diseases such as Parkinson’s disease [18]. Additionally, NF-1 inhibits the excessive production of reactive oxygen species (ROS) within cells following high glucose exposure, thereby reducing cell apoptosis. This makes NF-1 a potential therapeutic candidate for treating diabetic neuroinflammatory lesions [19]. NF-1 has recently been reported to upregulate the expression of TJ proteins such as ZO-1 [20]. In this study, the protective effects of NF-1 against Aβ_1-42_-induced senescence in BECs and disruption of the BBB were investigated to provide a potential therapeutic strategy for AD.

## Materials and methods

### Study design

For in vitro experiments, bEnd.3 cells were divided into four groups (control, Aβ1-42, Aβ1-42 + 30 nM NF-1, Aβ1-42 + 60 nM NF-1) with *n* = 6 wells per group for each assay (e.g., SA-β-gal staining, FITC-dextran, TEER, real-time PCR, Western blotting). Each well represented an independent biological replicate, seeded from a single stock (iCell, China). All in vitro experiments were independently repeated three times. For in vivo experiments, male and female Tg APPswe/PSEN1dE9 and WT mice (Jackson Laboratories, USA), aged 6–8 months, were divided into four groups: WT (*n* = 8), Tg (*n* = 8), Tg + 10 μg/kg NF-1 (*n* = 8), and Tg + 20 μg/kg NF-1 (*n* = 8). Each group included 4 males and 4 females to reflect the clinical prevalence of AD in both sexes and comply with NIH guidelines on sex as a biological variable [6]. The 6–8-month age range was chosen to capture significant amyloid plaque burden and BBB dysfunction, which are robust in Tg mice at this stage [8, 12]. Each mouse represented an independent biological replicate, with tissues harvested from the frontal cortex. In vivo experiments were performed once, as preliminary studies confirmed consistent BBB permeability changes in Tg mice [18]. Sample sizes were chosen based on prior studies detecting significant differences in cellular senescence and BBB permeability [10, 18]. No samples or animals were excluded, and all cells with ≥80% confluence and mice with confirmed Tg APPswe/PSEN1dE9 genotype were included, as per pre-established criteria. Mice were randomly allocated to groups using a random number generator, and investigators were blinded to group allocation during outcome assessments (e.g., SA-β-gal staining, FITC-dextran, TEER, immunostaining) to minimize bias.

### Cell culture, treatment, and transduction

bEnd.3 cells were purchased from iCell (China) and cultured in complete endothelial culture medium (ECM) under conditions of 37 °C and 5% CO_2_. bEnd.3 cells were authenticated by STR profiling within the last 6 months and tested negative for mycoplasma contamination using a PCR-based assay. Each well represented an independent biological replicate, seeded at 3 × 10⁵ cells per well for permeability assays or 2 × 10⁵ cells for telomerase activity (*n* = 6 per group). Experiments were repeated three times. To overexpress VEGF-R1, bEnd.3 cells were transduced with an adenovirus supplemented with the overexpression vector of VEGF-R1 (Ad-VEGF-R1) for 48 h. NF-1 concentrations of 30 and 60 nM were selected for in vitro experiments based on prior studies demonstrating effective modulation of cellular responses in neuronal and endothelial cells without toxicity [18, 20]. The 14-day treatment duration was chosen to reflect chronic Aβ_1-42_-induced senescence, consistent with established models [10].

### Animal experiments

Wild-type (WT) mice and Tg (APPswe/PSEN1dE9) AD model mice were purchased from Jackson Laboratories (USA). Each mouse represented an independent biological replicate (*n* = 8 per group). Each group included 4 males and 4 females to reflect the clinical prevalence of AD in both sexes and comply with NIH guidelines on sex as a biological variable [6]. Mice were divided into four groups: WT, Tg, Tg + 10 μg/kg NF-1, and Tg + 20 μg/kg NF-1. In the Tg + 10 μg/kg NF-1 and Tg + 20 μg/kg NF-1 groups, Tg mice were intraperitoneally injected with 10 μg/kg/day and 20 μg/kg/day NF-1 for 3 months. In the WT and Tg groups, WT and Tg mice were intraperitoneally injected with the same volume of normal saline for 3 months. All animal experiments complied with the ARRIVE guidelines and were carried out following the National Institutes of Health guide for the care and use of Laboratory animals. Animal experiments utilized NF-1 doses of 10 and 20 μg/kg/day, previously shown to be neuroprotective in mouse models [18]. The 3-month treatment duration was selected to correspond with the chronic AD pathology timeline in Tg APPswe/PSEN1dE9 mice. Animal experiments were approved by the Ethics Committee of the Seventh Affiliated Hospital of Sun Yat-sen University.

### SA-β-gal staining

SA-β-gal staining detects β-galactosidase activity at pH 6.0, a widely used marker of cellular senescence reflecting lysosomal changes in senescent cells. bEnd.3 cells (*n* = 6 wells per group) were fixed with 2 mL of 4% paraformaldehyde for 15 min. Subsequently, 1 mL of freshly prepared SA-β-Gal staining working solution was added. After sealing and incubating at 37 °C in a CO_2_-free incubator for 16 h in the dark, images were captured using a fluorescence microscope (Zeiss, Germany). Experiments were repeated three times.

### Measurement of telomerase activity

Telomerase activity and hTERT expression indicate telomere maintenance capacity, which is reduced in senescent cells, while TERF2 protects telomere ends, and its dysregulation is associated with senescence [21]. A total of 2 × 10^5^ bEnd.3 cells (*n* = 6 wells per group) were counted and collected, then resuspended in 200 μl of CHAPS lysis buffer (Takara, Japan) on ice for 30 min. Cells were centrifuged at 4 °C at 16,000 × g for 30 min. The supernatant was aliquoted and stored at −80 °C. Telomerase activity detection followed Voglauer’s qPCR method: a 20 μl reaction system included 1 μl of cell lysate (equivalent to 1 × 10^3^ cells), 0.5U Hotstar DNA polymerase (Takara, Japan), 200 nM TS primer (5’-AATCCGTCGAGAACAGTT-3’), 100 nM Cxa primer (5’-GTGTAACCCTAACCCTAACCC-3’), and 0.4×SYBR-Green I. The reaction conditions were as follows: incubation at room temperature for 30 min, denaturation at 95 °C for 5 min, followed by qPCR with 95 °C for 15 s and 50 °C for 60 s for 45 cycles. PCR was performed using the GeneAmp 9700 system (Thermo Fisher Scientific, USA). Experiments were repeated three times.

### Real-time PCR assay

Brain tissues from the frontal cortex of each mouse (*n* = 8 per group) were used for RNA and protein analyses, as this region is significantly affected in AD pathology. RNA was extracted from bEnd.3 cells (*n* = 6 wells per group) and brain tissues. Each sample was run in triplicate (technical replicates), with *n* = 6 wells per group for cells and *n* = 8 mice per group for tissues. The concentration and purity of the extracted RNA were measured and recorded using a nucleic acid detector (KAIAO, China), ensuring that the sample’s OD260/OD280 ratio was within the range of 1.8 to 2.0. Subsequently, first-strand cDNA was synthesized using the MonScript RT III All-in-One Mix with dsDNase (Monad Biotech, China). The PCR amplification mixture consisted of 1 μL each of first-strand cDNA, forward primer, and reverse primer, 10 μL of MonAmp 2× Taq Mix Pro (+Dye) (Monad Biotech, China), and 7 μL of ddH_2_O. The reaction protocol was as follows: initial denaturation at 94 °C for 3 min, followed by 25 cycles of denaturation at 94 °C for 15 s, annealing at 55 °C for 15 s, extension at 72 °C for 15 s, and a final extension at 72 °C for 5 min. Experiments were repeated three times.

### Western blotting assay

bEnd.3 cells (*n* = 6 wells per group) were lysed using RIPA cell lysis solution and PMSF solution (100 mM) at a ratio of 100:1. Further lysis was performed with an ultrasonic cell disruptor. The lysate was then centrifuged at 4 °C at 12,000 r·min-1 for 30 min, and the protein concentration was determined using the BCA method. The supernatant was taken, and an appropriate volume of protein loading buffer (diluted 4 times) was added. The mixture was denatured by heating in a 100 °C water bath for 10 min and then stored at −80 °C. SDS-PAGE and transfer were conducted, followed by a 1-h blocking with 5% non-fat milk. The diluted primary antibodies against p53, p21, VEGF-R1, VEGF-R2, ZO-1, claudin-5 (CST, USA, 1:1000), hTERT, TERF2 (Abcam, USA, 1:1000) and β-actin (CST, USA, 1:4000) were introduced, and the mixture was incubated for 12 h at 4 °C. After extensive washing with 1 × TBST, the diluted secondary antibody (CST, USA, 1:2000) was added, and the mixture was incubated for 2 h. Following further extensive washing with 1 × TBST, chemiluminescence was performed. Protein expression levels were detected using Image J software, with β-actin as an internal reference. Experiments were repeated three times.

### Measurement of FITC-dextran

bEnd.3 cells (*n* = 6 wells per group) were plated at a density of 3×10^5^ per well in a 12-well Transwell culture plate. When cells reached approximately 80% confluence, 1 mg/ml of FITC-Dextran was added to the upper chamber in a light-protected manner. The plate was then incubated in a cell culture incubator for 6 h. Subsequently, 200 μl of the culture medium from the lower chamber was aspirated, and the OD value at 492 nm was measured to construct a standard curve for FITC-Dextran. Based on this standard curve, the concentration of FITC-Dextran in the lower chamber of each well was calculated. Experiments were repeated three times.

### Transendothelial electric resistance (TEER) detection

TEER measures the electrical resistance across the endothelial monolayer, reflecting the integrity and permeability of the BBB, with lower values indicating barrier dysfunction. bEnd.3 cells (*n* = 6 wells per group) were seeded in a 12-well Transwell culture plate. After completing the drug treatment, the resistance of each well was measured using an electrical resistance meter (Sigma, Germany). During the measurement, the longer end of the electrode from the resistance meter was in contact with the bottom of the culture plate, while the shorter end was positioned near the bottom of the chamber. Three different locations within each chamber were selected for measurement, and the average value was taken as the actual TEER measured value. The resistance values were expressed in Ω·cm². The actual TEER was calculated by subtracting the blank value from the measured value and then multiplying by 1.12 cm². Experiments were repeated three times.

### Evans blue staining

Two hours before tissue collection, mice (*n* = 8 per group) in each experimental group were injected via the tail vein with 2% Evans Blue (EB) staining solution (4 mL/kg). After 2 h of systemic circulation, brains were harvested following cardiac perfusion and placed in a brain-slicing mold to prepare 2 mm continuous coronal brain sections. The areas of EB staining in the brains of mice were documented through photography, and their absorbance was measured using a microplate reader (MD, USA) to establish a standard curve. Based on the standard curve, the content of EB within the brain tissue of each experimental group (ng/mg protein) was calculated. Experiments were independently repeated twice, with results averaged to ensure reproducibility.

### Immunostaining assay

Paraffin sections were prepared from the frontal cortex of mouse brains (*n* = 8 per group). After deparaffinization, antigen retrieval, and blocking of endogenous peroxidase, the sections were incubated for 20 min. An appropriate number of primary antibodies against VEGF-R1, ZO-1, and claudin 5 (1:100, CST, USA) was then applied and allowed to incubate at 4 °C overnight. On the following day, after washing off the primary antibodies, secondary antibodies (TRITC conjugated secondary antibody was used for VEGF-R1 staining, 1:200, CST, USA) were applied and incubated for 1 h. For ZO-1 and claudin 5 staining, DAB chromogen solution was then added. The reaction was terminated when the cells were observed to turn brown-yellow under the microscope (Zeiss, Germany). The sections were then dehydrated, cleared, and mounted. The average optical density values were analyzed using Image J software. Experiments were independently repeated twice, with results averaged to ensure reproducibility.

### Statistical analysis

Data are expressed as mean ± standard deviation (SD), with error bars in all figures representing SD. A two-way ANOVA (sex × treatment) was used to assess potential sex differences in outcomes, with no significant sex interactions detected (*P* > 0.05). For sample sizes (*n* = 6 for in vitro experiments, *n* = 8 for in vivo experiments), one-way analysis of variance (ANOVA) with Tukey post hoc tests was used for multiple group comparisons. Data were tested for normality using the Shapiro-Wilk test and for homogeneity of variances using Levene’s test to confirm ANOVA assumptions.

All assays used sample sizes *n* ≥ 5 (*n* = 6 for in vitro, *n* = 8 for in vivo), therefore descriptive statistics (mean ± SD) were used to summarize the data. Tests were two-sided, and no adjustments for multiple comparisons were applied, as each figure tested a single primary hypothesis. *P*-values are reported in figure legends, with significance set at *P* < 0.05. Statistical analyses were performed using GraphPad Prism 8.0.2.

## Results

### NF-1 reduced cellular senescence in bEnd.3 cells exposed to oligomerized Aβ_1-42_

**Fig. 1:**
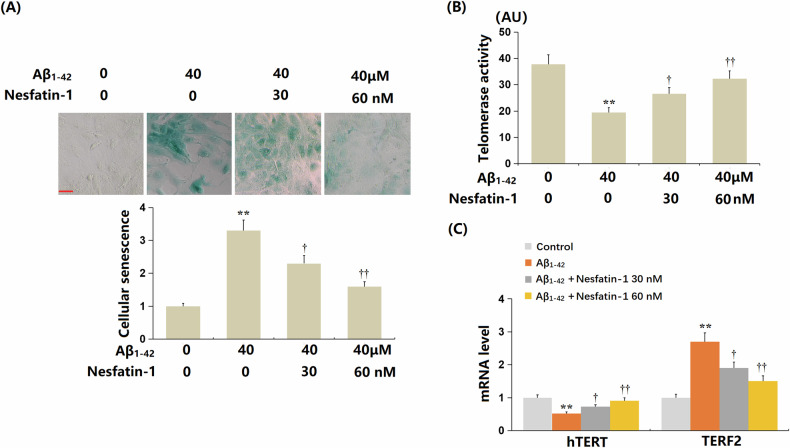
Nesfatin-1 ameliorated cellular senescence of brain endothelial cells against oligomerized Aβ_1-42_ in bEnd.3 brain endothelial cells. Cells were stimulated with oligomerized Aβ_1-42_ (40 μM) with or without Nesfatin-1 (30, 60 nM) for 14 days. **A** Cellular senescence was assayed using SA-β-gal staining. Scale bar, 50 μm; (**B**) Telomerase activity. **C** The mRNA level of *hTERT* and *TERF2*. Data represent mean ± SD from three independent experiments; *n* = 6 wells per group per experiment. **P < 0.01 vs. vehicle group; ^†^, ^††^*P* < 0.05^,^ 0.01 vs. Aβ_1-42_ treatment group (one-way ANOVA with Tukey post hoc test).

**Fig. 2:**
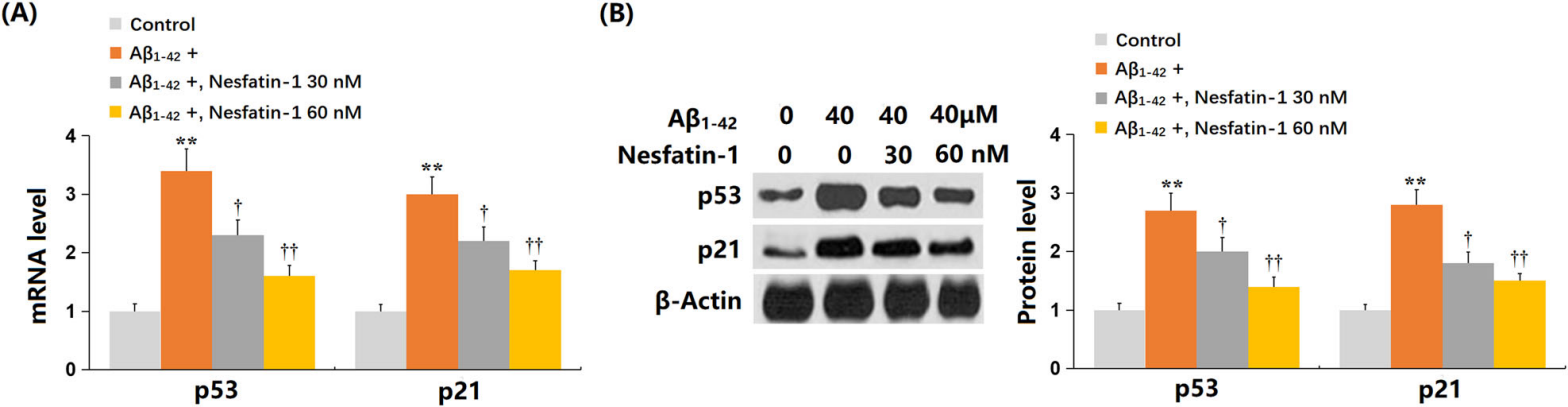
Nesfatin-1 reduced the expression of p53 and p21 against Aβ_1-42_ in bEnd.3 brain endothelial cells. Cells were stimulated with oligomerized Aβ_1-42_ (40 μM) with or without Nesfatin-1 (30, 60 nM). **A** The mRNA expression of *p53* and *p21* as measured by real-time PCR. **B**. Protein of p53 and p21 as measured by western blot analysis. Data represent mean ± SD from three independent experiments; *n* = 6 wells per group per experiment. **P < 0.01 vs. vehicle group; ^†^, ^††^*P* < 0.05^,^ 0.01 vs. Aβ_1-42_ treatment group (one-way ANOVA with Tukey post hoc test).

bEnd.3 cells (*n* = 6 wells per group) were exposed to oligomerized Aβ_1-42_ (40 μM) in the presence or absence of NF-1 (30, 60 nM) for 14 days. The proportion of SA-β-gal stained cells significantly increased upon Aβ_1-42_ treatment, but this increase was sharply reduced by both 30 and 60 nM NF-1 (Fig. 1A). Telomerase activity in Aβ_1-42_-challenged bEnd.3 cells significantly decreased from 37.8 AU to 19.5 AU; however, it was markedly restored to 26.6 AU and 32.3 AU by 30 nM and 60 nM NF-1, respectively (Fig. 1B). Additionally, the reduction in hTERT and upregulation of TERF2 observed in Aβ_1-42_-treated bEnd.3 cells were strikingly reversed by 30 and 60 nM NF-1 (Fig. 1B). p21 and p53 are important biomarkers of cellular senescence. Levels of p53 and p21 were considerably upregulated in Aβ_1-42_-challenged bEnd.3 cells, but this upregulation was markedly suppressed by 30 and 60 nM NF-1 (Fig. 2A, B).

### NF-1 inhibited the oligomerized Aβ_1-42_-induced increase in VEGF-R1 levels but did not affect VEGF-R2 level in bEnd.3 cells

**Fig. 3:**
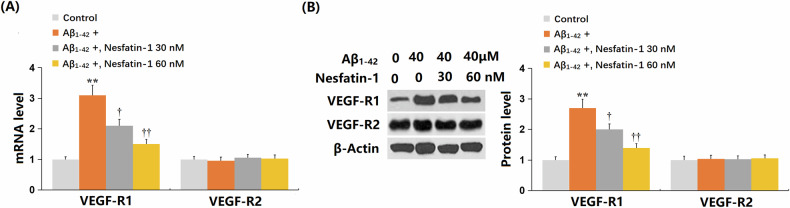
Nesfatin-1 suppressed the expression of VEGF-R1 induced by oligomerized Aβ_1-42_ but not that of VEGF-R2 in bEnd.3 brain endothelial cells. Cells were stimulated with oligomerized Aβ_1-42_ (40 μM) with or without Nesfatin-1 (30, 60 nM). **A** The mRNA levels of *VEGF-R1* and *VEGF-R2* as measured by real-time PCR. **B** Protein levels of VEGF-R1 and VEGF-R2 as measured by western blot analysis. Data represent mean ± SD from three independent experiments; *n* = 6 wells per group per experiment. **P < 0.01 vs. vehicle group; ^†^, ^††^*P* < 0.05^,^ 0.01 vs. Aβ1-42 treatment group (one-way ANOVA with Tukey post hoc test).

Both VEGF-R1 [10] and VEGF-R2 [22] are known to be involved in cellular senescence. In this study, Aβ_1-42_ significantly elevated VEGF-R1 expression in bEnd.3 cells, which was notably reduced by treatment with 30 and 60 nM of NF-1. However, there was no significant difference in VEGF-R2 expression among the four groups (Fig. 3A, B). These findings suggest that VEGF-R1, rather than VEGF-R2, may play a role in the impact of NF-1 on Aβ_1-42_-induced senescence in bEnd.3 cells.

### Overexpression of VEGF-R1 abrogated the beneficial effects of NF-1 against oligomerized Aβ_1-42_ induced cellular senescence in bEnd.3 cells

**Fig. 4:**
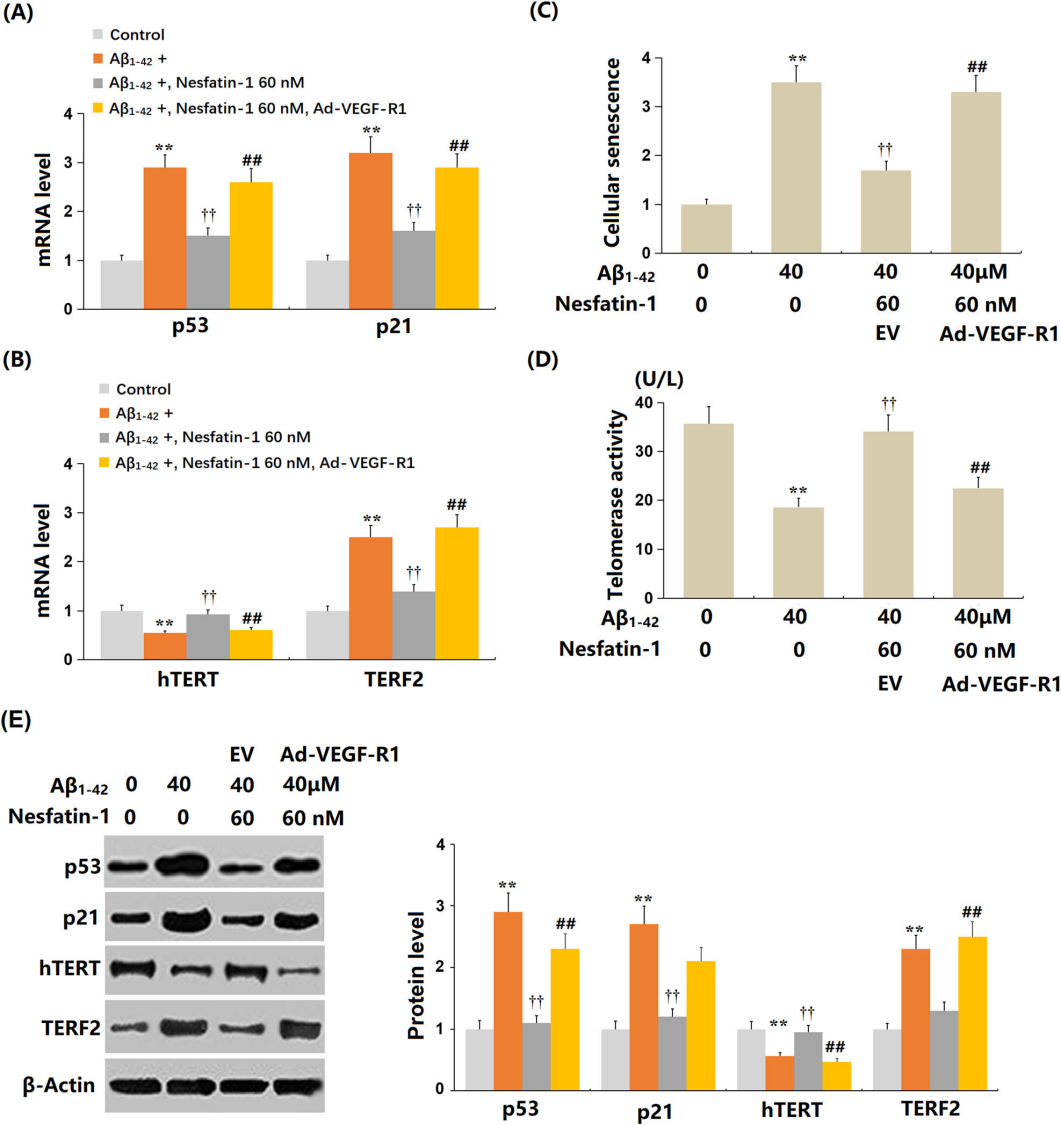
Overexpression of VEGF-R1 abrogated the beneficial effects of Nesfatin-1 against cellular senescence induced by oligomerized Aβ_1-42_ in bEnd.3 brain endothelial cells. Cells were transduced with Ad-VEGF-R1, followed by stimulation with oligomerized Aβ_1-42_ (40 μM) with or without Nesfatin-1 (60 nM). **A** The mRNA levels of *p53* and *p21*. **B** The mRNA levels of *hTERT* and *TERF2*. **C** Cellular senescence was assayed using SA-β-gal staining. **D** Telomerase activity (**P < 0.01 vs. vehicle group; †, ††*P* < 0.05, 0.01 vs. Aβ_1-42_ treatment group). **E** Western blotting measured p53, p21, hTERT, and TERF2 levels in Ad-VEGF-R1-bEnd.3 cells treated with Aβ_1-42_ (40 μM) alone or concomitantly with Nesfatin-1 (60 nM). Data represent mean ± SD from three independent experiments; *n* = 6 wells per group per experiment. **P < 0.01 vs. vehicle; ^†^*P* < 0.05 vs. Aβ_1-42_; ^#^*P* < 0.05 vs. A^β^_1-42_ + Nesfatin-1 (one-way ANOVA with Tukey post hoc test).

To validate the involvement of VEGF-R1, cells (*n* = 6 wells per group) were transduced with Ad-VEGF-R1 and then treated with oligomerized Aβ_1-42_ (40 μM) in the presence or absence of NF-1 (60 nM). The enhanced mRNA levels of *p53* and *p21* in Aβ_1-42_-stimulated bEnd.3 cells were substantially restrained by NF-1 but were markedly boosted by VEGF-R1 overexpression (Fig. 4A). Moreover, *hTERT* mRNA was downregulated, and *TERF2* mRNA was upregulated in Aβ_1-42_-stimulated bEnd.3 cells, which were visibly reversed by NF-1. However, following VEGF-R1 overexpression, *hTERT* mRNA levels notably decreased, and *TERF2* mRNA levels strikingly increased (Fig. 4B). Furthermore, the increased number of SA-β-gal-stained bEnd.3 cells induced by Aβ_1-42_ was noticeably reduced by NF-1 but considerably increased by VEGF-R1 overexpression (Fig. 4C). The telomerase activity in the control, Aβ_1-42_, Aβ_1-42_ + NF-1, and Aβ_1-42_ + NF-1+ Ad-VEGF-R1 groups was 35.7, 18.6, 34.1, and 22.5 AU, respectively (Fig. 4D). The protein levels of p53, p21, hTERT, and TERF2 were measured by Western blotting. Aβ_1-42_ treatment significantly increased p53 and p21 protein levels while reducing hTERT and increasing TERF2. NF-1 (60 nM) markedly reduced p53 and p21 and restored hTERT and TERF2 levels. Overexpression of VEGF-R1 reversed these effects, increasing p53 and p21 and altering hTERT and TERF2 levels (Fig. 4E). These results collectively demonstrate that VEGF-R1 overexpression counteracts NF-1’s protective effects against Aβ_1-42_-induced cellular senescence in bEnd.3 cells, confirming VEGF-R1’s critical role in mediating NF-1’s actions.

### NF-1 alleviated the aggravation of endothelial monolayer permeability induced by oligomerized Aβ_1-42_

**Fig. 5:**
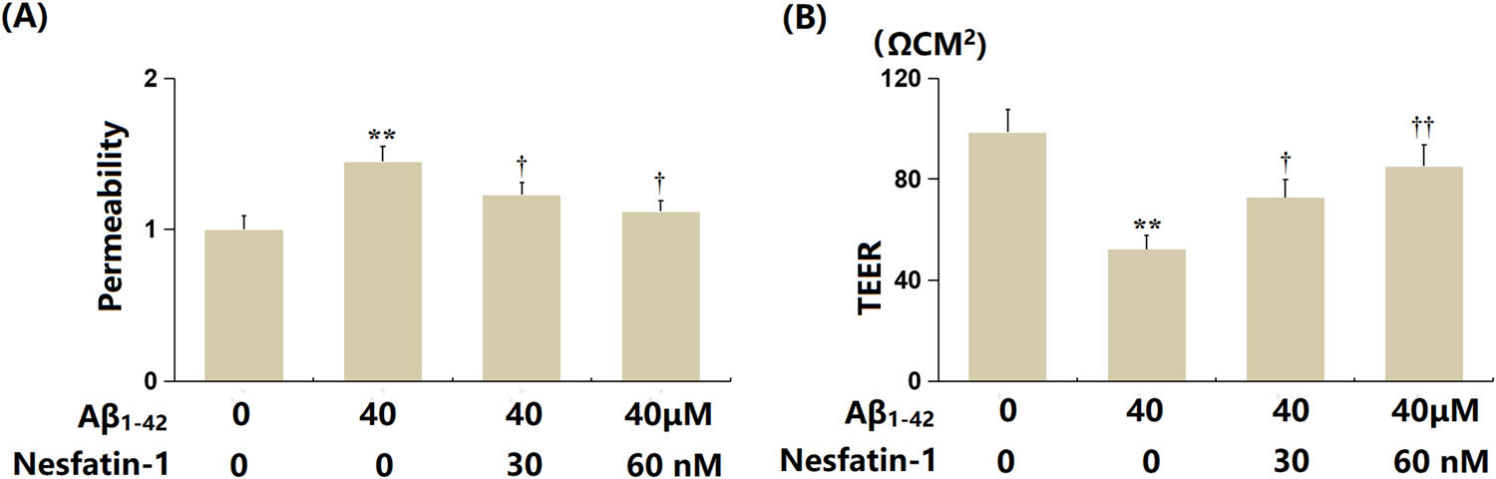
Nesfatin-1 alleviated the oligomerized Aβ_1-42_-induced aggravation of endothelial monolayer permeability. Cells were stimulated with oligomerized Aβ_1-42_ (40 μM) with or without Nesfatin-1 (30, 60 nM). **A** Permeability of the bEnd.3 monolayer was measured using the FITC-dextran across assay. **B** Trans-endothelial electrical resistance (TEER) was assayed. Data represent mean ± SD from three independent experiments; *n* = 6 wells per group per experiment. **P < 0.01 vs. vehicle group; ^†^, ^††^*P* < 0.05^,^ 0.01 vs. Aβ1-42 treatment group (one-way ANOVA with Tukey post hoc test).

bEnd.3 cells (*n* = 6 wells per group) were treated with oligomerized Aβ_1-42_ (40 μM) in the presence or absence of NF-1 (30, 60 nM). The increased FITC fluorescence in Aβ_1-42_-treated bEnd.3 cells was markedly reduced by NF-1 at 30 and 60 nM concentrations (Fig. 5A). TEER values in Aβ_1-42_-treated bEnd.3 cells notably declined from 98.6 to 52.5 Ωcm², which were considerably boosted to 72.8 and 85.1 Ωcm² by 30 and 60 nM NF-1, respectively (Fig. 5B).

### NF-1 restored the expression of ZO-1 and claudin-5 in bEnd.3 cells

**Fig. 6:**
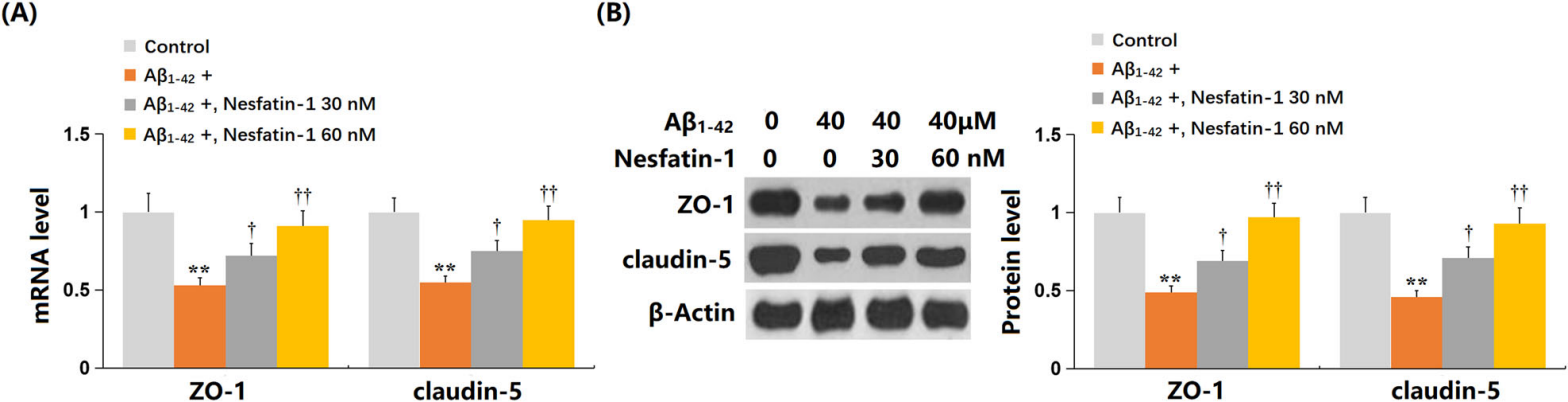
Nesfatin-1 restored the expression of ZO-1 and claudin-5 in bEnd.3 brain endothelial cells. Cells were treated with oligomerized Aβ_1-42_ (40 μM) in the presence or absence of Nesfatin-1 (30, 60 nM). **A** The mRNA levels of *ZO-1* and *claudin-5*. **B** Protein levels of ZO-1 and claudin-5. Data represent mean ± SD from three independent experiments; *n* = 6 wells per group per experiment. **P < 0.01 vs. vehicle group; ^†^, ^††^*P* < 0.05^,^ 0.01 vs. Aβ1-42 treatment group (one-way ANOVA with Tukey post hoc test).

ZO-1 and claudin-5 are crucial TJ proteins that constitute the BBB [23]. In bEnd.3 cells challenged with Aβ_1-42_, the levels of ZO-1 and claudin-5 were significantly downregulated. However, treatment with 30 and 60 nM NF-1 markedly enhanced the expression of these TJ proteins (Fig. 6A, B).

### Overexpression of VEGF-R1 abrogated the protective effects of NF-1 against oligomerized Aβ_1-42_-induced endothelial monolayer permeability in bEnd.3 cells

**Fig. 7:**
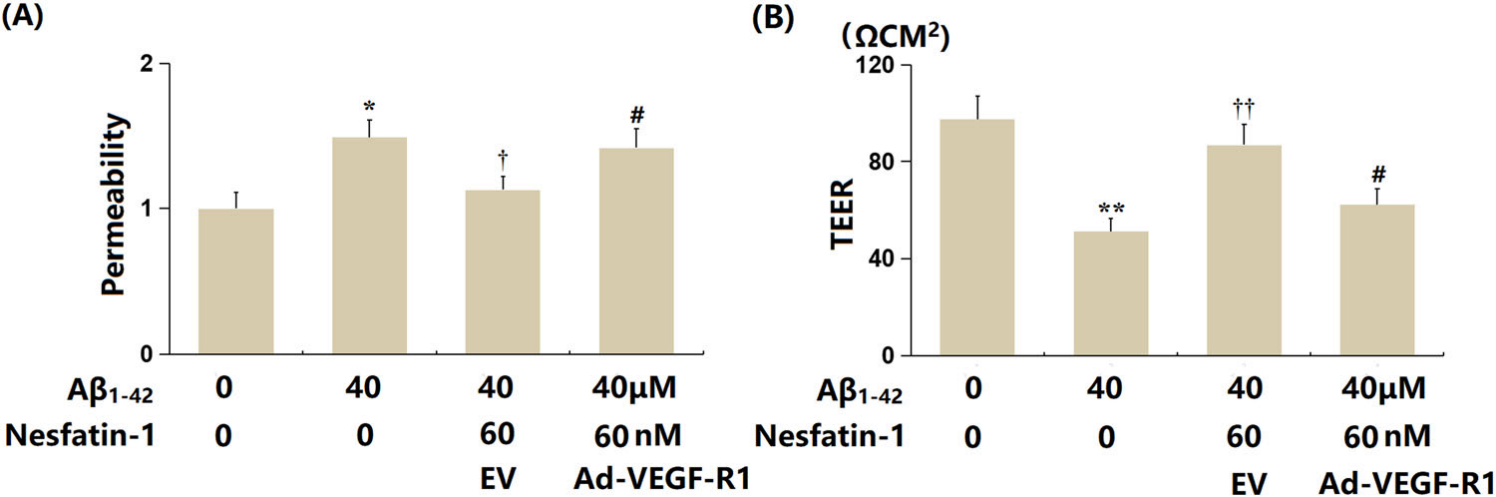
Overexpression of VEGF-R1 abrogated the protective effects of Nesfatin-1 against oligomerized Aβ_1-42_-induced aggravation of endothelial monolayer permeability in bEnd.3 brain endothelial cells. **A** Permeability of the bEnd.3 monolayer was measured using the FITC-dextran across assay. **B** Trans-endothelial electrical resistance (TEER) was assayed. Data represent mean ± SD from three independent experiments; *n* = 6 wells per group per experiment. *, **P < 0.05, 0.01 vs. vehicle group; ^†^, ^††^*P* < 0.05^,^ 0.01 vs. Aβ_1-42_ treatment group; ^#^*P* < 0.05 vs. Aβ_1-42_ + Nesfatin-1 group (one-way ANOVA with Tukey post hoc test).

Cells (*n* = 6 wells per group) were transduced with Ad-VEGF-R1 and then treated with oligomerized Aβ_1-42_ (40 μM) in the presence or absence of NF-1 (60 nM). The increased FITC fluorescence intensity observed in Aβ_1-42_-treated cells was significantly reduced by NF-1, but this reduction was counteracted by overexpression of VEGF-R1 (Fig. 7A). Additionally, the TEER value, which dropped from 97.5 to 51.3 Ωcm² in Aβ_1-42_-treated cells, was restored to 86.9 Ωcm² by NF-1 treatment. However, following VEGF-R1 overexpression, the TEER value decreased sharply to 62.3 Ωcm² (Fig. 7B).

### The administration of NF-1 reduced the expression of VEGF-R1 in the brain cortex of AD mice

**Fig. 8:**
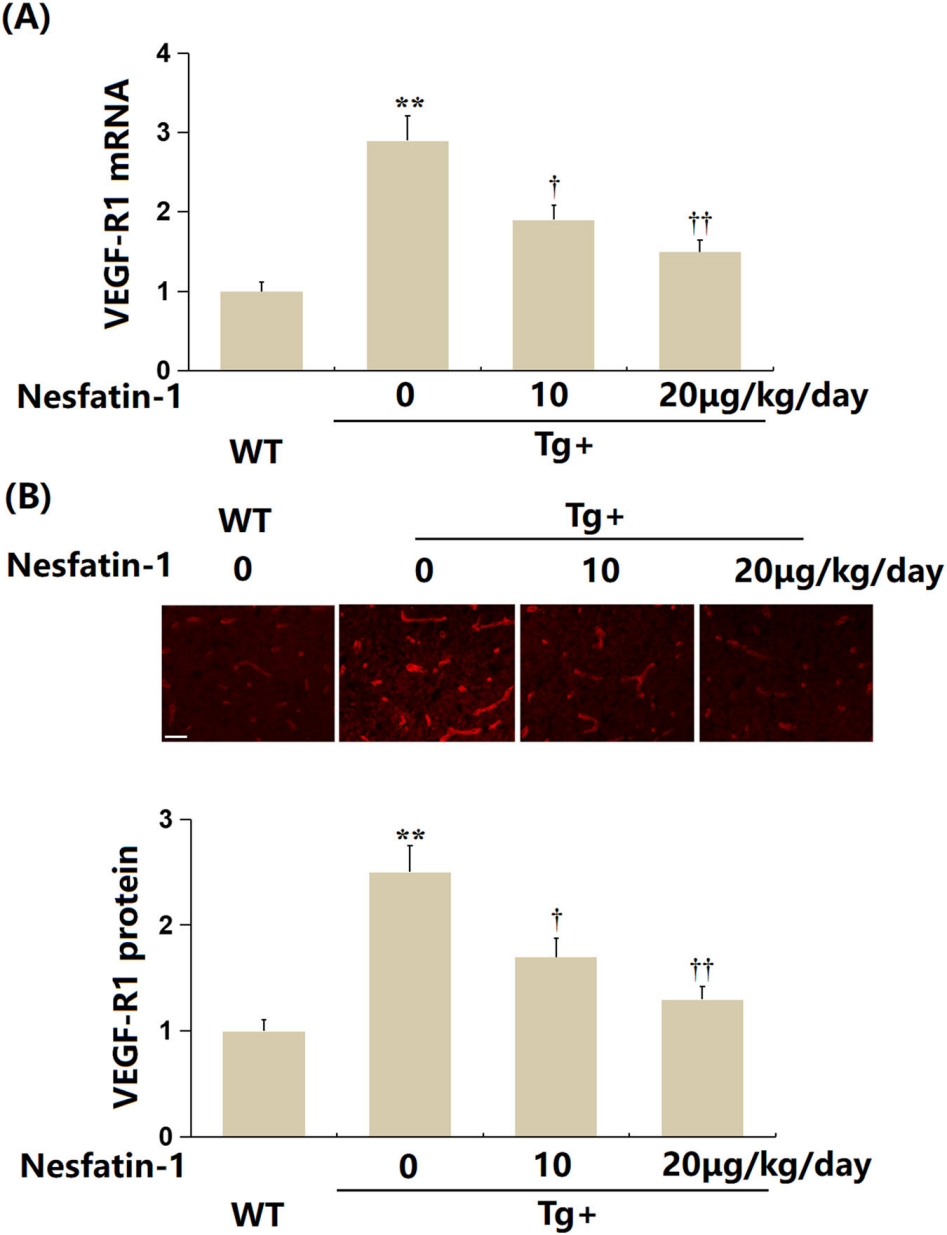
Administration of Nesfatin-1 reduced the expression of VEGF-R1 in the brain cortex of Alzheimer’s disease (AD) mice. Mice were divided into four groups: wild-type (WT) mice, AβPPswe/PSEN1dE9 transgenic (Tg) mice, Tg+ Nesfatin-1 at 10 μg/kg/day for 3 months, and Tg+ Nesfatin-1 at 20 μg/kg/day for 3 months. **A** The mRNA levels of *VEGF-R1*. **B** Protein levels of VEGF-R1 as measured by immunostaining. Scale bar, 100 μm. Data represent mean ± SD; *n* = 8 mice per group (4 males, 4 females). **P < 0.01 vs. WT group; ^†^, ^††^*P* < 0.05^,^ 0.01 vs. Tg, Tg + Nesfatin-1 10 μg/kg/day group (one-way ANOVA with Tukey post hoc test).

To investigate the effect of NF-1 on BBB integrity in AD mice, animals (*n* = 8 per group) were divided into four groups: wild type (WT), transgenic (Tg), Tg + 10 μg/kg NF-1, and Tg + 20 μg/kg NF-1. VEGF-R1 levels were significantly elevated in the Tg group but were markedly decreased following treatment with both 10 and 20 μg/kg NF-1 (Fig. 8A, B).

### The administration of NF-1 alleviated BBB dysfunction in the brains of AD mice

**Fig. 9:**
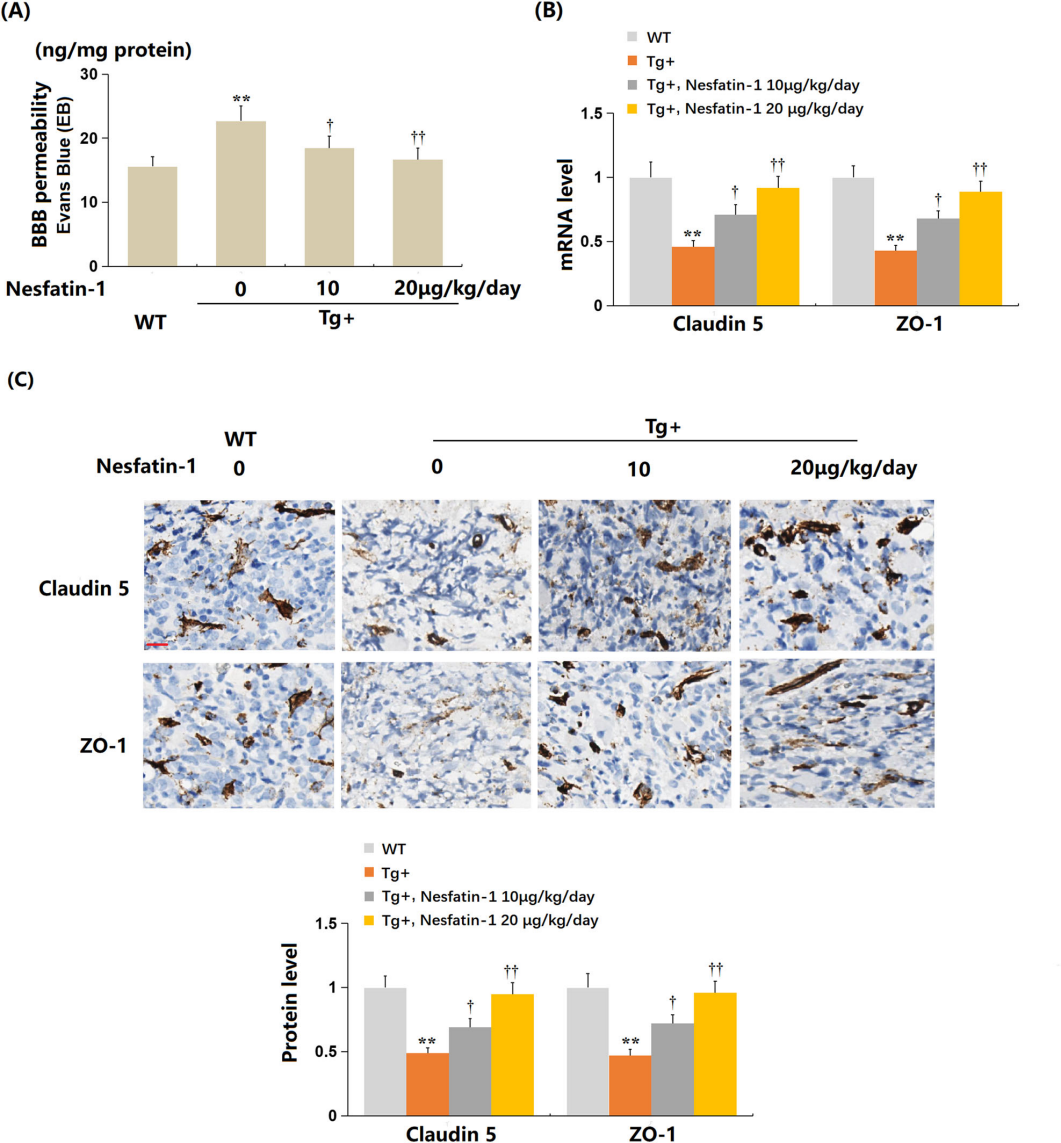
Administration of Nesfatin-1 alleviated blood-brain barrier (BBB) dysfunction in the brains of Alzheimer’s disease (AD) mice. Mice were divided into four groups: wild-type (WT) mice, AβPPswe/PSEN1dE9 transgenic (Tg) mice, Tg+ Nesfatin-1 at a dose of 10 μg/kg/day for 3 months, and Tg+ Nesfatin-1 at a dose of 20 μg/kg/day for 3 months. **A** BBB permeability was measured by quantifying Evans Blue dye content (ng/mg protein) in brain tissue, indicating the extent of dye leakage across the BBB. **B** The mRNA levels of *claudin 5* and *ZO-1*. **C** protein levels of claudin 5 and ZO-1 as measured by immunostaining. Scale bar, 100 μm. Data represent mean ± SD; *n* = 8 mice per group (4 males, 4 females). **P < 0.01 vs. WT group; ^†^, ^††^*P* < 0.05^,^ 0.01 vs. Tg, Tg + Nesfatin-1 10 μg/kg/day group (one-way ANOVA with Tukey post hoc test).

In Tg mice, the content of Evans Blue (EB) within the brain tissue increased sharply from 15.6 to 22.7 ng/mg protein. However, treatment with 10 and 20 μg/kg NF-1 significantly reduced the levels to 18.5 and 16.7 ng/mg protein, respectively (Fig. 9A). Additionally, the notably suppressed expressions of ZO-1 and claudin-5 observed in Tg mice were substantially restored by both 10 and 20 μg/kg NF-1 treatments (Fig. 9B, C).

## Discussion

Previous studies have suggested a neuroprotective role for NF-1 in neurodegenerative diseases. For instance, Shen et al. demonstrated that NF-1 protects dopaminergic neurons against MPP + /MPTP-induced toxicity in Parkinson’s disease models via the C-Raf-ERK1/2 pathway [18]. Similarly, NF-1 reduces oxidative stress and apoptosis in neuronal cells under high-glucose conditions, indicating its potential in diabetic neuropathy [19]. However, its role in AD, particularly in protecting the BBB, has not been extensively investigated. Our study builds on these findings by demonstrating that NF-1 mitigates Aβ_1-42_-induced endothelial senescence and BBB dysfunction through VEGF-R1 regulation, a novel mechanism not previously reported. These positions NF-1 as a potential therapeutic agent for AD, addressing a critical gap in targeting BBB integrity to alleviate AD pathology.

BECs are the key cell types involved in the constitution of the BBB. They regulate the exchange of substances and intercellular communication by forming TJs, thereby maintaining the stability of the brain environment [24]. As age progresses, these BECs may undergo senescence, a process that significantly impacts the function of the BBB. Senescent BECs can lead to BBB dysfunction, characterized by compromised integrity of intercellular junctions and decreased expression of TJ proteins. This BBB dysfunction allows substances from the bloodstream to more easily permeate into brain tissue, altering the intracranial environment and potentially leading to the occurrence of neuroinflammation and neurodegenerative diseases [11]. Herein, similar to the results reported by Park [25], the permeability of the bEnd.3 monolayer was sharply increased by Aβ_1-42_, which was significantly inhibited by NF-1, suggesting a protective role of NF-1 against Aβ_1-42_-triggered endothelial monolayer permeability enlargement. Furthermore, the repair effect of NF-1 on the Aβ_1-42_-induced BBB disruption was further validated in AD mice. Moreover, in line with data presented by Angom [10], severe cellular senescence was notably induced by Aβ_1-42_ in BECs, which was considerably alleviated by NF-1. This suggests that NF-1 might protect BBB integrity by alleviating cellular senescence in BECs.

Our study shows that NF-1 counters Aβ_1-42_-driven cellular aging and BBB damage by lowering VEGF-R1 levels, but the exact mechanisms by which NF-1 influences VEGF-R1 is still unknown, since no specific receptor for NF-1 has been found [13]. We speculate that NF-1 might work indirectly, possibly by adjusting pathways that link to VEGF-R1 control. For example, earlier research indicates NF-1 activates the C-Raf-ERK1/2 route in dopamine neurons, which may affect VEGF-R1 transcription or related signaling components [18]. Also, NF-1’s ability to decrease oxidative damage and inflammation [19] could reduce VEGF-R1 expression indirectly, as these stressors are known to increase VEGF-R1 activity [26]. Another possibility is that NF-1 alters VEGF-R1 through connections with upstream molecules, like VEGF-A or other growth factors, but this needs more study. The loss of NF-1’s protective effects on cellular aging and endothelial barrier function after VEGF-R1 overexpression (Figs. 4 and 7) highlights VEGF-R1’s key role in NF-1’s actions, though whether this involves direct receptor contact or indirect pathways is still unclear. Future work to identify the NF-1 receptor and explore its signaling routes will be crucial to understand how NF-1 regulates VEGF-R1 and affects tight junction proteins (ZO-1 and claudin-5) and aging markers (p53 and p21). These efforts will boost NF-1’s potential as a treatment for AD by clarifying its mechanism.

ZO-1, an important TJ protein, is widely present in various epithelial and endothelial cells and is considered crucial for maintaining the structure and function of intercellular TJs. By interacting with other TJ proteins on the cell membrane, such as occludin and claudins, ZO-1 forms a complex network that helps seal the intercellular spaces, thereby preventing the non-selective permeation of extracellular substances [27, 28]. Claudin-5, a TJ protein belonging to the claudin family, is primarily expressed in vascular endothelial cells and plays an essential role in maintaining the integrity and selective permeability of the BBB. Working in concert with other TJ proteins, such as ZO-1, claudin-5 forms a tight network of intercellular junctions that effectively controls and regulates paracellular transport of water molecules, ions, and small molecules while preventing the disorderly permeation of large molecules and cells [29, 30]. Herein, parallel to the reports by Chan et al. [31] and Wu et al. [32], downregulation of ZO-1 and claudin-5 was observed in both Aβ_1-42_-stimulated bEnd.3 cells and the brain cortex of AD mice. These proteins were noticeably upregulated by NF-1, implying that NF-1 might protect BBB integrity by maintaining a TJ structure.

VEGF-R1 is recognized as an important cell surface receptor and a member of the VEGF family. It plays a pivotal role in angiogenesis, lymphangiogenesis, and the regulation of vascular permeability. Its intrinsic tyrosine kinase activity is activated by binding to various ligands within the VEGF family, particularly VEGF-A. This activation triggers a series of signal transduction events that promote the proliferation, migration, and tubule formation of endothelial cells [26, 33]. Recent research indicates that VEGF-R1 is involved in the regulation of the senescence process of endothelial cells [34]. Herein, it was found that VEGF-R1 was sharply upregulated in Aβ_1-42_-treated bEnd.3 cells and its levels were remarkably reduced by NF-1. This indicates that NF-1 might alleviate Aβ_1-42_-triggered senescence in BECs by restraining VEGF-R1 expression. Furthermore, the influence of NF-1 on cellular senescence in bEnd.3 cells was substantially abolished by VEGF-R1 overexpression. This validated that NF-1 mitigated Aβ_1-42_-induced senescence in BECs by downregulating VEGF-R1. Moreover, in Aβ_1-42_-stimulated bEnd.3 cells, the facilitating effect of NF-1 on the expressions of TJ proteins was abrogated by VEGF-R1 overexpression. This suggests that NF-1 maintained TJ structure in Aβ_1-42_-treated BECs by repressing VEGF-R1 signaling. Furthermore, in the brain cortex of AD mice, the upregulation of TJ proteins following NF-1 treatment was accompanied by declined VEGF-R1 expression. This further identified that VEGF-R1 is a key regulator in NF-1-induced BBB protection. Our future studies will further confirm the influence of NF-1 on behavioral changes in AD mice to verify the therapeutic potential of NF-1 against AD.

Collectively, NF-1 protected BBB integrity in AD by preventing cellular senescence of BECs and enhancing the expression of ZO-1 and claudin-5, mediated through the regulation of VEGF-R1. Our findings indicate that NF-1 has the potential to be a therapeutic agent for treating AD.

## Conclusion

This study indicates that NF-1 may contribute to reducing Aβ_1-42_-induced cellular aging and blood-brain barrier impairment in bEnd.3 cells and AD mice by potentially decreasing VEGF-R1 levels and supporting the expression of tight junction proteins ZO-1 and claudin-5. These observations suggest that NF-1 could play a role in protecting BBB integrity in AD, potentially offering a promising avenue for therapeutic exploration. Additional research is required to further elucidate its mechanisms and evaluate its therapeutic potential.

## Data Availability

The data of this study will be made available from the corresponding authors on request. No protein, DNA, RNA sequences, macromolecular structures, crystallographic data, or microarray data were generated, negating the need for public repository deposition.
